# How is caring for grandchildren associated with grandparents’ health: the mediating effect of internet use

**DOI:** 10.3389/fpubh.2023.1196234

**Published:** 2023-08-09

**Authors:** Jie Wang, Rongxing Gu, Lanxi Zhang, Li Zhang

**Affiliations:** ^1^School of Law, Anhui Normal University, Wuhu, Anhui, China; ^2^School of Sociology, China University of Political Science and Law, Beijing, China; ^3^Department of Sociology and Anthropology, School of Oriental and African Studies, University of London, London, United Kingdom; ^4^School of Sociology, China University of Political Science and Law, Beijing, China

**Keywords:** internet use, grandparental childcare, health, China Health and Retirement Longitudinal Study (CHARLS), mediating effect, the Karlson-Holm-Breen (KHB) method

## Abstract

**Objective:**

Prior studies showed mixed results of the association between grandchild care and grandparents’ health. This research focused on the mechanisms behind the above link by studying how internet use served as a mediator through which grandchild care has impacted grandparents’ health. The study aimed to draw implications to improve health of grandparents who offer care to grandchildren.

**Methods:**

Using a sample of 16,829 grandparents aged 50 through 80 from the 2018 wave of China Health and Retirement Longitudinal Study (CHARLS), the study relied on the KHB method to conduct the analysis. Grandparental health was measured by self-rated health (SRH), instrumental activity of daily living (IADL), life satisfaction and depression.

**Results:**

Overall, grandchild care had a positive effect on grandparental health. Those who engaged in grandchild care were more likely to use internet. In addition, internet use mediated the ways in which grandchild care impacted grandparents’ health. Interne use generally promoted the positive influence of grandparental caregiving on grandparents’ health. Specifically, the mediating effects of watching videos and chatting through the internet were most pronounced among urban grandmothers. The mediating effects of watching news were most noticeable among both urban grandmothers and grandfathers.

**Conclusion:**

Internet use served as a mediator in the association between grandchild child care and grandparental health. Promoting internet usage may be an effective way reducing the negative impact of grandchild care on grandparents’ mental health. It could also increase the positive effect of caregiving on grandparents’ SRH and functional independence. The study also underscored the importance of taking rural–urban context and gender role into consideration when studying intergenerational caregiving and Chinese grandparents’ health.

## 1. Introduction

Grandparents have been found to play a vital role in caring for grandchildren, especially in traditional Asian societies where highly value multigenerational co-residence as an ideal living arrangement. Such a living arrangement pattern is considered as a form which promotes filial piety and collective family interests (1). Multigenerational co-residence also makes the involvement of grandchild care be on a daily basis without stating a custodial responsibility (2, 3). In recent years, with a rising trend of female labor force participation and an increasing divorce rate, grandparents’ involvement in childbearing becomes even more important. Consequently, considerable research attention has been given to studies of grandparental childcare and its impacts on grandparents’ mental and physical health. Mixed results have been found with showing a positive, negative, U-shape or non-significant impact of grand parenting on grandparental health. Some studies have attempted to explore the mechanisms that may explain the ways in which caring for grandchildren links to grandparents’ health status. They indicated that family cohesion, intergenerational relations, social supporting networks and participating in social activities mediated the relationship between grandparental caregiving and grandparents’ health (4, 5). Prior literature, however, has hardly examined the mediating effect of internet use in such a linkage. This study tried to fill the voids of prior literature by relying on the Karison, Holm and Breen (KHB) method to analyze data taken from the 2018 wave of China Health and Retirement Longitudinal Study (CHARLS).

When examining the mediating effect, this study also concerned gender as well as rural–urban differences. This is because previous research highlighted significant gender differences in China’s traditional family norms in terms of domestic life and caregiving practice (6, 7). Studies reported that grandmothers spent a comparable amount of time in childcare, which was three times as much time as grandfathers spent in childcare (8). When caring for grandchildren, Chinese grandmothers tended to fulfill intensive responsibilities, such as feeding, bathing, and dressing whereas grandfathers usually served as fun-seeker, playmate, and companion (9). Grandfathers may also experience social stigma if they are heavily involved in grandparenting since traditional Chinese norms do not encourage males to be heavily engaged in childcare, which deviates from the cultural norm and the masculine and dominant role of males in a family. In Chinese culture, males are considered as breadwinners and they are not supposed to be involved in caregiving. Thus, scholars indicated that grandfathers may have acquired fewer skills, resources, and coping strategies to offer appropriately care to both their parents and grandchildren (10). Previous literature reported worse psychological health among grandmothers than grandmothers (11). Studies based on Western countries also showed significant gender differentials. For instance, a positive association was found between grandchild care and grandmothers’ physical health but was not found among grandfathers (12). More depressive symptoms were reported for grandmothers than for grandfathers (13, 14). The effect of social media reducing depression was also found to be more apparent among older women than among older men (15).

Drastic rural–urban differences in social resources, cultural tradition, living standards, and individual health disparities have been heavily documented in prior literature as well (16–20). The family caregiving norms have also been found to vary in rural and urban China (21). Although traditional family norms and cultural values have been largely weakened in recent decades by China’s rapid urbanization and dramatic social changes, less changes have occurred in rural areas as compared to urban settings (22). With a large number of rural residents moving to cities to seek job opportunities, rural Chinese grandparents are more likely than their urban peers to become custodial caregivers in skipped-generation households. Rural grandparents are more financially dependent on their adult children and consider grandchild care as a reciprocal form of intergenerational exchange (23). As a result, rural grandparents’ psychological well-being tends to be more impacted by both financial security and grandchild caregiving. Since the one-child policy was more strictly implemented in urban than in rural China, urban grandparents have less caregiving burden. They tend to enjoy nurturing the only grandchild in family when rural grandparents are struggling to care multiple grandchildren. These differences are likely to cause grandparental health disparities due to grandchild care giving.

Under such a proceeding, when investigating the mediating effects of internet use in the linkage of grandparent caregiving and grandparental health, the paper also aimed to examine whether the mediating effects of internet use vary by grandparents’ gender and rural–urban residence. Thus, internet use was the only mediator but grandparents were broken down into subgroups based on their gender and residence to show group variation. This study contributed to the existing literature in several ways. First, the paper systematically investigated the mediating effects of internet use when breaking down grandparents into subgroups based on their gender and rural–urban residence, which has been rarely seen in previous literature. Second, unlike most prior analyzes of Chinese grandparenting that relied on regional, non-representative samples, this study estimated publicly available data from a nationally representative longitudinal survey, which yielded more generalizable and replicable findings. Third, when measuring health outcomes, unlike many previous analyses that restricted to grandparents’ psychological well-being, this study examined health in a more comprehensive manner by also including physical health measures, i.e., self-reported health status and functional limitations. Below we review prior literature that guided this empirical work.

## 2. Literature review

### 2.1. Grandchild care and grandparental health

Previous studies have found negative, positive and even U shape associations between grandchild care and grandparental health. Multiple theories can be used to explain such findings. The *role conflict theory* argued that the custodial role of caring for grandchildren conflicts with grandparents’ other roles. This is because grandparenting is time consuming and is physically and psychologically demanding for grandparents. It is generally associated with a decrease in time available for hanging out with friends and doing recreational activities. Some grandparents may have given up employment to raise grandchildren. Custodial grandmothers have been found to be more likely economically disadvantaged because job and income loss increased their economic vulnerability and psychological stress (24). The *Social isolation theory* further contended that lacking of privacy, social engagement and leisure time led to heightened isolation and depression (25), which brought consequently negative effects on grandparents’ health (26). In addition, the stress of caregiving could also cause or exacerbate poor health behaviors, such as smoking (27). Some custodial grandmothers were even reported to have increased psychological distress due to child behavior problems (28).

Several other theories, however, explained a positive link between grandparenting and grandparents’ health. For instance, the *healthy lifestyle theory* highlighted that caring for a grandchild may have resulted in a more active lifestyle, healthier meals, or a reduction in smoking (27, 29). It has made grandparents stay active and has prevented elders from deteriorating at older ages (30). Caring grandchildren could also stimulate the brain, which was helpful in maintaining good cognitive skills (31). Meanwhile, grandparenting have enhanced grandparents’ self-esteem, self-worth as well as family cohesion (12, 32). Grandchildren could be great emotional resources for grandparents, which led to reduced depressive symptoms (33) and greater life satisfaction (34, 35). Caregiving to grandchildren was also found to be able to reduce grandparents’ mortality rate (36). The intergenerational *time-for-money exchange theory* also supported a positive association between the amount of grandchild care provided by grandparents and the amount of remittance received from their migrant adult children. Such a reciprocal exchange improved grandparents’ living standard and health care, which in turn benefited their physical health, reduced depressive symptoms and improved level of life satisfaction (37).

Still others showed no significant association or a nonlinear relationship between grandparenting and grandparental health. Arpino & Bordone (30) found that except for verbal frequency, providing child care did not show statistically significant effects on other domains of grandparents’ cognitive function. Hughes et al. (38) suggested that the health disadvantages among grandparent caregivers arose from grandparents’ prior characteristics, not as a consequence of providing care. Oshio’s (39) study suggested that caring for grandchildren did not have either a beneficial or detrimental effects on grandparents’ health. Other scholars supported a non-linear relationship between giving care to grandchildren and grandparents’ health. For instance, Glaser et al. (40) claimed that non-intensive levels of caregiving were most likely to be associated with better health. Grandparents who provided custodial childcare and grandparents who did not provide any grandchild care were likely to report poor health.

The inconsistent findings on caring for grandchildren and grandparental health shown in the existing literature can largely be explained by the following: (1) methodological issues, (2) different social-cultural background since grandparenting types and responsibilities vary by country, policy, and culture, and (3) the studying subjects. In short, whether the studied grandparents were primary caregivers and the amount of time grandparents spent on caregiving affected research results. Primary caregivers tended to be more involved in child care, as a result, they were more likely to report worse health outcomes.

### 2.2. Internet use and individual health

Prior literature has showed a strong link between internet use and individual health. The *social causation theory* emphasized the crucial roles social conditions within people’s networks play in influencing one’s mental health (41). Studies showed that the effects of internet use on depressive symptoms among older adults were mediated by increasing social engagement levels to reduce the levels of loneliness, create contact with social ties and connect with social support (42–44). Social engagement through internet use benefited seniors’ cognitive function and subjective well-being (45). The *compensatory internet use theory argued* that online networks built by internet use were able to provide seniors various forms of support and entertainment which may not be obtained from daily life (46, 47). Regarding physical health, studies found that internet and smart phone usage helped older adults in terms of health maintenance and chronic disease self-management, communication with professionals, monitoring health status, and receiving health promotion interventions (48, 49). Using internet and smart phones have been documented as supporting daily life activities by functioning as navigators and memory aids, which preserved seniors’ independence and improved quality of life (45).

Excessive or compulsory using of internet, however, was found to be associated with decreased time spent with friends and a lower level of local social networking, which increased loneliness and lower quality of life (50). Problematic social media use was found to be linked to reduced social and psychological well-being (51). In addition, internet use decreased in-person and face-to-face social activities, which resulted in a lack of personal contacts (46). Scholars found that heavier Facebook users tended to believe that other people were happier and had better lives (52). The *social comparison theory* contended that such kind of passive internet use may lead to upwards social comparison and envy, bring psychological loss and relative deprivation, which caused depressive mood and reduced life satisfaction (53, 54).

### 2.3. Caring for grandchildren, internet use and grandparents’ health

Previous studies documented that social support networks mediated the association between grandparenting and grandparents’ health. This is because social networks can play a significant role in helping individuals to cope with adverse situations. By analyzing the Chinese sample, Tang et al. (55) found that the association between grandchild care and depressive symptoms among grandparents was fully mediated by their social networks. They contended that grandparents who took care of grandchildren tended to have larger social networks, and giving care to grandchildren could reduce depressive symptoms through having larger social networks. Zhou and associates (56) illustrated that intergenerational support, especially emotional support, played a mediating role in the relationship between caregiving stress and grandparental health. Similar findings were also documented in literature studying sample from Western countries. Through studying grandparents in Spain, it was found that social support networks along with personal character strengths acted as protective factors that mediated the link between amount of care provided to grandchildren and grandparents’ health related quality of life (57).

The use of internet has been found to be strongly related to older adults’ social networks. Internet using was found to be able to enlarge older adults’ social networks and increase the level of social support, including intergenerational support. Studies examining the Western social context showed that using internet increased social contact between seniors and other people, which helped older adults receiving more support from their adult children and others. Consequently, it promoted elders’ health status (58, 59). Studies of Chinese elders echoed such findings. It was documented that internet use extended social networks of the older adults by improving the level of their social participation (60). Other scholars also provided evidence that using internet increased intergenerational contact, which helped Chinese older adults receiving more intergenerational support from their adult children (61, 62).

Given findings of previous studies that social support networks mediated the association between grandparenting and grandparental health, and internet use increased elders’ social networks, we hypothesized that internet use can possibly mediate the link between grandparent caregiving and grandparental health. Thus, this study attempted to elucidate whether and how using internet helps grandparent caregivers to achieve better health status. By taking gender and rural–urban differences into consideration, the findings clarified the role of internet use in protecting against caregiving stress and negative influence among male and female grandparents and among rural and urban residents in China. The study aimed to advance our understanding of health advantages of internet use among grandparent caregivers.

## 3. Materials and methods

### 3.1. Data

Data were drawn from the 2018 survey of the China Health and Retirement Longitudinal Study (CHARLS), a nationally representative survey of adults aged 45 and older and their spouses. The CHARLS was conducted by the China Center for Economic Research of Peking University. Detailed information on demographic characteristics, social and economic conditions, and health and health-related behaviors was collected based on face-to-face interviews in respondents’ homes. Using a multi-stage probability-proportional-to-size technique, participants were randomly sampled by regions and then by urban districts or rural counties and *per capita* GDP. In total, CHARLS sampled residents from 150 counties across 28 provinces in China. This study focused on individuals aged 50 through 80 who were grandparents and provided care to their grandchildren. In this study, 16,829 respondents (8,060 males and 8,769 females) aged 50 to 80 were taken from the 2018 CHARLS survey. Among them, 12,080 were rural and the rest 4,769 were urban.

### 3.2. Variables

In terms of the variables applied in the analysis, the dependent variables were mental and physical health of grandparents. Although previous studies have applied a striking array of health outcome measures, these measures can largely be classified into four dimensions: (a) mortality, morbidity and frailty, including chronic illnesses (63, 64), (b) perceived health or self-rated health (65, 66), (c) functional health which is indicated as ADL and recurrent falling (67, 68), (d) mental health, including physiological wellbeing, depression, life satisfaction and cognitive function (69–71). The CHARLS questionnaire did have questions asking about older adults’ self-rated health, IADL, life satisfaction as well as depression. These measures are consistent with the above four dimensions of commonly used health outcome measures. Thus, they should be able to capture the health status of the respondent.

In this research, *mental health* was measured by two continuous variables: life satisfaction and depressive symptoms, both of which have been used as validate measures of psychological well-being in existing literature (11). Life satisfaction variable was obtained from the survey question: “Please think about your life-as-a-whole. How satisfied are you with it?” A 5-pointcoding scale was applied for the responses ranging from 1 (“not at all satisfied”) to 5 (“completely satisfied”). Depressive symptoms were measured by the 10-item Center for Epidemiologic Studies Depression Scale (CESD-10), which has been repeatedly used in prior literature as a measure of depression (16, 72). The depressive symptoms were captured by 10 questions asking if the respondent: 1) was bothered by things, 2) had trouble keeping mind on what he/she was doing, 3) felt depressed, 4) felt everything he/she did was an effort, 5) felt hopeful about the future, 6) felt fearful, 7) felt that sleep was restless, 8) was happy, 9) felt lonely, and 10) could not get “going.” The depression symptom measure used in this research was obtained by summing the above CESD-10 scores, which ranged from 0 to 40, with higher values indicating more depressive symptoms.

*Physical health* was captured by self-rated health (SRH) and difficulty in instrumental activities of daily living (IADLs). The IADLs included managing money, taking medications, grocery shopping, preparing meals, making phone calls and cleaning the house. If the respondent had no difficulty with the above six activities, then he or she was coded as “1,” “2” if had some difficulty but could still made it, “3” if had difficulty and needed help, and “4” if the respondent could not complete the above activities. Consequently, the IADL score ranged from 6 to 24. Those who had difficulty with one of these above activities was considered as having IADL difficulties. The SRH score ranged from 1 to 5 with “1” representing “very poor health” and “5” representing “excellent health.” Both variables were continuous with higher scores indicating a greater difficulty in instrumental activities of daily living and a better self-rated health condition, respectively.

There were two key independent variables capturing grandparental childcare. One was grandparents’ self-reported grandchild caregiving experience in the past year. If those who reported giving care to one or more grandchildren, then the respondent was coded as “1” and “0” if otherwise. The other independent variable asked that during the past year, one average, number of hours the respondent or his/her spouse took care of grandchild(ren) per day, which was coded as a continuous variable.

The mediating variables were taken from three survey questions in CHARLS, which asked the respondent’s internet usage. The three questions asked if the respondent used internet to chat, watch videos, or watch news, respectively. The three mediating variables were coded as dichotomous ones with “1” indicating “yes” and “0” if otherwise.

The control variables included age, educational attainment, annual household *per capita* income, number of adult children, having weekly contact with adult children (1 = yes, 0 = no). Annual household *per capita* income was generated from annual household income which was summarized from self-reported multiple income sources (e.g., salary and wage, capital income, pension, government transfer, and other unspecified sources) and divided by number of household members. The socioeconomic variables were controlled because socio-economic condition has been shown to have an effect on one’s later-life health status and mortality (73, 74).

### 3.3. Methods

The analyses began with descriptive results that provided initial insights into the descriptive information of all variables included in the research (see Table 1). The study then applied the KHB method, a recently developed mediation approach, to determine the extent to which the selected mediating variables explained the total effect of grandparent caregiving on grandparental health. The KHB method provided an unbiased decomposition of total effects into direct and indirect effects for nonlinear probability models (75, 76). It allowed for the comparison of odds by subtracting residuals of the variables that were subsequently added in the original equation. In other words, the method allowed us to examine mediation effects in logistic probability models. The estimates of these models were more robust than standard logit models (77).

**Table 1:** Descriptive statistics of variables: Chinese grandparents aged 50–80 (%).

Variables	All	Cared for at least 1 grandchild past year	Did not care for grandchildren past year
**Dependent variables**			
*Mental health*			
CES-D score(mean)**	18.23	18.18	18.28
<22 (no depressive symptom) ***	67.76	69.00	66.75
≥22 (had depressive symptoms) ***	32.24	31.00	33.25
Life satisfaction**			
Completely satisfied	4.99	4.91	5.05
Very satisfied	28.81	29.37	28.34
Somewhat satisfied	54.68	55.17	54.27
Not very satisfied	8.42	7.81	8.92
Not at all satisfied	3.11	2.74	3.41
*Physical health*			
IADL(mean)***	7.22	7.03	7.39
If had IADL difficulties**			
Yes	26.12	25.04	27.00
No	73.88	74.96	73.00
Self-rated health			
Very good	12.49	12.17	12.76
Good	12.89	12.65	13.08
Fair	49.25	50.67	48.07
Poor	19.63	19.24	19.95
Very poor	5.75	5.27	6.14
**Independent variables**			
Number of hours cared for grandchild(ren)/day (mean)	–	6.69	–
<3 h/day	–	39.85	–
3 ≤ x < 6 h/day		17.02	
6 ≤ x < 12 h/day	–	26.48	–
≥12 h/day	–	16.65	–
**Mediating variables**			
If used internet to chat***			
Yes	9.38	10.19	8.38
No	90.62	89.81	91.62
If used internet to watch news***			
Yes	11.38	12.53	9.96
No	88.62	87.47	90.04
If used internet to watch videos***			
Yes	9.26	10.00	8.35
No	90.74	90.00	91.65
**Control variables**			
Age(mean)	63.99	64.02	63.96
Sex**			
Male	47.89	47.13	48.51
Female	52.11	52.87	51.49
Residence			
Urban	28.22	28.70	27.83
Rural	71.78	71.30	72.17
Educational attainment			
Illiterate	22.69	19.29	19.40
Elementary school	42.81	43.51	44.07
Middle and high school	32.33	35.94	33.88
College and above	2.17	1.26	2.65
If lived with spouse			
Yes	81.36	83.87	79.32
No	18.64	16.13	20.68
Household *per capita* income(mean)	5,055.73	3,608.13	6,498.21
If received pension			
Yes	86.46	87.50	85.63
No	13.54	12.50	14.37
Number of chronic diseases(mean)	0.71	0.71	0.72
*N*	16,829	7,541	9,288

2018 China Health and Retirement Longitudinal Study (CHARLS).

*p < 0.05; **p < 0.01; ***p < 0.001 represent significance levels when conducting Chi-square or T-tests to check sample differences between those who cared for grandchildren and those who did not.

**Figure 1:**
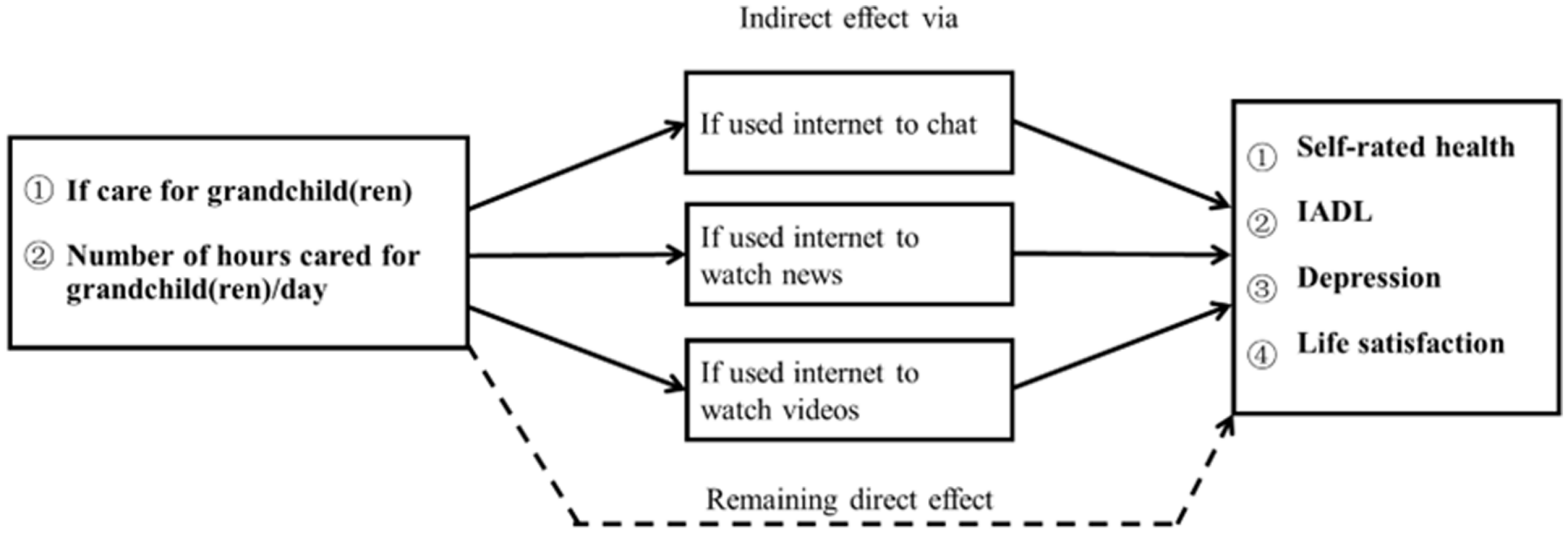
Potential mechanisms linking grandparental childcare to grandparents’ health.

The total effect represented the impact of caring for grandchildren on grandparental health without including mediating variables as controls. The direct effect was obtained by using a full model with mediators, and indicated the (remaining) effect of caring for grandchildren on grandparental health (unexplained part). The indirect effects represent the parts of the total effect that ran through different mediating variables. The analyzes were performed by using a user-written KHB module for Stata (76). In this study, age, education and other control variables were covariates. Since we were interested in exploring the rural–urban as well as gender differences, sample in our study were divided into four subgroups and all the models were fitted to each of the four group subsample (urban men, rural men, urban women, and rural women). The Chi-square tests and T-tests were applied to test whether the between group differences were statistically significantly different for dichotomous and continuous variables, respectively. The potential mechanisms linking grandparental child care to grandparents’ health outcomes were presented in Figure 1.

## 4. Results

### 4.1. Descriptive statistics

Table 1 summarized the descriptive information of the variables applied in the analysis. The average depressive symptom CES-D scores for respondents who had and who had not grandchild caregiving experience during the past year were 18.18 and 18.28, respectively. A CES-D score ≥ 12 for a CES-D range of 0 to 30 has been commonly used as the cut-off of having depressive symptoms (71). Since this study coded CES-D from 10 to 40, we applied 22 as the cut-off point. When applying this cut off point, the results showed that 32.2% of the respondents had depressive symptoms. The percentage of respondents who had depressive symptoms was lower among grandparents who had grandchild care experience (31.0%) than those who did not care for grandchild(ren) (33.3%). The group difference was statistically significant, indicating that mental health status for grandparents with grandchild caregiving experience was significantly better than that of their counterparts with no such experience. The majority of the respondents reported very satisfied or somewhat satisfied with their life. Higher percentages of respondents who did not care for grandchildren reported “not very satisfied” or “not at all satisfied” with life. Such a difference was statistically significant, which again showed that giving care to grandchildren improved grandparents’ life satisfaction. As to physical health, the mean IADL score for those who did not care for grandchildren was higher than that of those who cared for grandchildren (7.39 vs. 7.03), with a *p* value lower than 0.01 when performing a t-test. When IADL was treated as a dichotomous variable, about 73.9% of grandparents had no IADL difficulties, and such percentage was higher for grandparents with grandchild caregiving experience. As to SRH status, the majority of the respondents rated their health as “fair.”

Regarding grandparental care giving variables, the results indicated that the average number of hours caring for grandchildren per day was 6.69. The majority of them reported cared grandchild(ren) for less than 3 h or between 6 to 12 h per day (39.9 and 26.5%, respectively). Those who cared for grandchild(ren) for 3 to 6 h and more than 12 h per day counted for 17.0 and 16.7%, respectively (see Table 1).

Grandparents who cared for grandchildren during the past year tended to report higher percentages of internet use for all three internet use (mediating) variables than their counterparts with no grandchild caregiving experience. Specifically, 10.2, 12.5 and 10.0% of the grandparents who cared for grandchildren reported used internet to chat, to watch news and to watch videos, respectively. And the corresponding percentages for non-caregivers were 8.4, 10.0 and 9.4%, respectively. According to the Chi-square test, the differences between these two subgroups were statistically significant.

The average age for studied respondents was 64 years old. The caregiver subgroup contained a lower percentage of males as compared to the non-caregiver subgroup (47.1% vs. 48.5%). Such a difference was statistically significant. Most respondents had low educational attainments. The average annual household *per capita* income was 5,055.7RMB (about 802 USD), with urban grandparents’ *per capita* annual income doubled that of rural grandparents. About 81.4% of the sample lived with a spouse and such a percentage was slightly higher for the subgroup with grandchild caregiving experience (83.9% vs. 79.3%). Over 86.0% of the respondents claimed they were pension receivers. The average number of chronic diseases reported by the respondents was below 1, indicating a fairly good health condition of the studied sample.

Since the study was interested in exploring the urban–rural as well as gender differences, the respondents who cared for grandchildren in the past year were further classified into four subsamples (urban men, rural men, urban women, and rural women) and the descriptive information for these subgroups was presented in Table 2. Disparities were shown in both mental and physical health outcomes among four subgroups. For instance, the average depressive symptom score was highest for rural grandmothers (19.6), followed by urban grandmothers (17.7), rural grandfathers (17.6) and urban grandfathers (16.2). When the CES-D cut off score of 22 was applied, the percentages of grandparents who had depression symptoms were higher among both rural grandfathers and grandmothers as compared to their urban counterparts. In general, the percentage of respondents who reported “not very satisfied” and “not at all satisfied” were higher among rural grandparents (especially grandmothers) as compared to their urban counterparts. As to physical health conditions, disparities in daily activity functional limitations were smaller. On average, urban grandfathers appeared to be the healthiest whereas rural grandmothers experienced the highest level of disability. Regarding SRH, higher percentages of urban grandparents tended to rate their health as “very good” or “good” than rural grandparents. Rural grandmothers were the group that had the highest percentages of respondents who rated their health as “poor” or “very poor” (24.9 and 6.1%).

**Table 2:** Descriptive statistics of variables for four subgroups: Chinese grandparents who cared for grandchildren in past year, aged 50–80 (%).

Variables	Grandfathers		Grandmothers	
	Urban	Rural	Urban	Rural
**Dependent variables**				
*Mental health*				
CES-D score (mean)	16.19	17.55	17.65	19.62
CES-D < 22 (no depressive symptoms)	77.27	73.17	70.66	61.62
CES-D ≥ 22 (had depressive symptoms)	22.73	26.83	29.34	38.38
Life satisfaction				
Completely satisfied	5.55	4.58	4.70	5.07
Very satisfied	25.72	29.49	28.94	30.67
Somewhat satisfied	61.58	56.75	57.79	50.55
Not very satisfied	5.34	7.39	6.36	9.59
Not at all satisfied	1.81	1.78	2.21	4.12
*Physical health*				
IADL(mean)	6.57	6.92	6.77	7.39
If R had IADL difficulties				
Yes	13.93	21.36	19.70	34.46
No	86.07	78.64	80.30	65.54
Self-rated health				
Very good	13.22	13.45	11.36	11.01
Good	18.87	12.34	14.56	10.06
Fair	48.89	50.41	55.49	49.26
Poor	14.29	18.22	14.74	23.61
Very poor	3.73	5.59	3.85	6.07
**Independent variables**				
Number of hours cared for grandchild(ren)/day (mean)	6.42	6.66	6.21	6.60
< 3 h/day	39.36	41.37	40.49	38.41
3 ≤ x < 6 h/day	18.29	16.18	19.11	16.45
6 ≤ x < 12 h/day	26.74	25.67	25.33	27.57
≥12 h/day	15.61	16.78	15.07	17.57
**Mediating variables**				
If used internet to chat				
Yes	13.74	6.69	15.71	5.01
No	86.26	93.31	84.29	94.99
If used internet to watch news				
Yes	22.73	9.21	15.45	3.84
No	77.27	90.79	84.55	96.16
If used internet to watch videos				
Yes	16.60	7.32	14.24	3.95
No	83.40	92.68	86.76	96.05
**Control variables**				
Age(mean)	65.25	64.43	64.25	63.13
Educational attainment				
Illiterate	2.96	9.32	16.41	35.24
Elementary school	32.91	48.66	35.77	45.82
Middle and high school	35.08	29.46	29.08	15.52
College and above	29.05	12.55	18.75	3.42
If lived with spouse				
Yes	86.07	89.18	77.08	81.09
No	13.93	10.82	22.92	18.91
Household *per capita* income(mean)	6331.25	3404.75	4765.46	2243.52
If received pension				
Yes	89.53	87.88	86.72	86.74
No	10.47	12.12	13.28	13.26
Number of chronic diseases(mean)	0.82	0.66	0.77	0.69
*N*	1,012	2,542	1,152	2,835

2018 China Health and Retirement Longitudinal Study (CHARLS).

Differentials were also observed in terms of internet usage. Around 15.0% of urban grandfathers and 15.0% of grandmothers reported using internet to chat and watch videos. About 22.7% of urban grandfathers and 15.5% of urban grandmothers used internet to watch news, respectively. In contrast, the percentage of rural grandparents who used internet to chat, watch news and watch videos ranged between 6.7 to 9.2%. Such percentages were even lower for rural grandmothers. The average age of studied grandparents ranged between 63.1 and 65.2 years old. Both rural–urban gaps and gender differences were evident in socioeconomic status. About 64.1% of urban grandfathers completed secondary education (middle school or above), which was more than three times as high as that for rural grandmothers (18.9%). The average annual household *per capita* income was 6,331.2 RMB (944.9 USD) and 4,765.5 RMB (711.2 USD) for urban grandfathers and grandmothers, respectively, which doubled the corresponding values for their rural counterparts. Urban grandfathers and grandmothers reported greater average numbers of chronic diseases than their rural counterparts. Over 86.0% of rural and urban grandparents received pension with minor differences. Percent of grandfathers living with spouses were much higher than grandmothers with little rural–urban differences.

### 4.2. Regression results

The next steps of the analysis was to detect: 1) whether the total effects of key grandchild caregiving variables, i.e., grandchild caregiving experience and number of hours cared for grandchild per day, on the respondent’s health outcomes were significant (see results of Model 1 in Tables 3, 4), 2) whether the effects of grandparenting acted through a given set of internet use mediators, which were the indirect impacts. When adding mediators in Model 2, if the values or the significance levels the regression coefficients presented in Model 1 changed, then it indicated the indirect effects existed (see results in Tables 3, 4), and 3) whether the ways in which grandchild caregiving impacted grandparents’ health were explained by these mediators (direct impact).

**Table 3:** OLS regression analysis of grandparental childcare on grandparents’ health outcomes: Chinese grandparents aged 50–80.

Variables	Self-Rated Health		IADL		Depression		Life Satisfaction	
	Model 1	Model 2	Model 1	Model 2	Model 1	Model 2	Model 1	Model 2
*Independent variable*								
If R Cared for grandchild(ren)	0.03^+^ (0.02)	0.02^+^ (0.02)	−0.42^***^ (0.47)	−0.32^***^ (0.47)	−0.20^+^ (0.11)	−0.18^+^ (0.11)	0.03^**^ (0.01)	0.03^**^ (0.01)
*Control variables*								
Age	−0.01^***^ (0.00)	−0.01^***^ (0.00)	0.07^***^ (0.00)	0.06^***^ (0.00)	−0.01 (0.01)	−0.02^*^ (0.01)	0.01^***^ (0.01)	0.01^***^ (0.01)
Sex	0.11^***^ (0.02)	0.11^***^ (0.02)	−0.38^***^ (0.05)	−0.38^***^ (0.05)	−1.66^***^ (0.11)	−1.64^***^ (0.11)	0.05^***^ (0.01)	0.05^***^ (0.01)
Urban residence	0.06^***^ (0.02)	0.05^*^ (0.02)	−0.28^***^ (0.06)	−0.25^***^ (0.06)	−1.11^***^ (0.13)	−1.00^***^ (0.13)	0.03 (0.02)	0.03 (0.02)
Educational attainment	0.15^***^ (0.02)	0.13^***^ (0.02)	−0.39^***^ (0.05)	−0.35^***^ (0.05)	−1.16^***^ (0.12)	−1.02^***^ (0.12)	−0.02 (0.01)	−0.02 (0.01)
If lived with spouse	0.06^**^ (0.02)	0.06^**^ (0.02)	−0.08 (0.06)	−0.09 (0.06)	−1.33^***^ (0.14)	−1.33^***^ (0.14)	0.12^***^ (0.02)	0.12^***^ (0.02)
Annual household *per capita* income	0.07^***^ (0.01)	0.06^***^ (0.01)	−0.19^***^ (0.02)	−0.19^***^ (0.02)	−0.39^***^ (0.04)	−0.37^***^ (0.04)	0.02^***^ (0.00)	0.02^***^ (0.00)
If received pension	−0.06^*^ (0.03)	−0.06^*^ (0.03)	−0.08 (0.07)	−0.07 (0.07)	0.06 (0.17)	0.08 (0.17)	0.04^*^ (0.02)	0.04^*^ (0.02)
# of chronic diseases	−0.25^***^ (0.01)	−0.25^***^ (0.01)	0.36^***^ (0.02)	0.36^***^ (0.02)	0.98^***^ (0.05)	0.98^***^ (0.05)	0.06^***^ (0.02)	0.06^***^ (0.01)
*Mediating variables*								
Used internet to chat		0.02 (0.04)		−0.25^*^ (0.12)		−0.31 (0.27)		0.00 (0.03)
Used internet to watch news		0.07 (0.04)		−0.12 (0.12)		−0.81^**^ (0.28)		−0.01 (0.04)
Used internet to watch videos		0.11^*^ (0.02)		−0.10 (0.13)		0.01 (0.29)		0.01 (0.04)
Constant	2.73^***^ (0.09)	2.77^***^ (0.09)	5.27^***^ (0.26)	5.36^***^ (0.26)	24.69^***^ (0.61)	24.89^***^ (0.04)	3.42^***^ (0.08)	3.42^***^ (0.08)
*N*	13,483	13,483	14,214	14,214	13,337	13,337	13,420	13,420
Adjusted R^2^	0.12	0.13	0.11	0.11	0.10	0.11	0.02	0.02

2018 China Health and Retirement Longitudinal Study (CHARLS).

^+^*p* < 0.1, ^*^p < 0.05, ^**^p < 0.01, ^***^p < 0.001. Numbers in parentheses are standard errors.

**Table 4:** OLS regression analysis of grandparental childcare length on grandparents’ health outcomes: Chinese grandparents aged 50–80.

Variables	Depression	
	Model 1	Model 2
*Independent variable*		
# of hours cared for grandchild(ren)/day	0.04^**^(0.01)	0.03^**^ (0.01)
*Control variables*		
Age	−0.03^*^ (0.01)	−0.03^**^(0.01)
Sex	−1.41^***^ (0.17)	−1.39^***^ (0.17)
Urban residence	−1.02^***^ (0.19)	−0.92^***^ (0.19)
Educational attainment	−1.10^***^ (0.17)	−0.95^***^ (0.18)
If lived with spouse	−0.92^***^ (0.22)	−0.93^***^ (0.22)
Annual household *per capita* income	−0.43^***^ (0.05)	−0.37^***^ (0.04)
If received pension	−0.08 (0.26)	−0.04 (0.26)
# of chronic diseases	0.81^***^ (0.08)	0.82^***^ (0.08)
*Mediating variables*		
Used internet to chat		−0.52 (0.42)
Used internet to watch news		−0.84^**^(0.44)
Used internet to watch videos		−0.04 (0.45)
Constant	25.48^***^ (0.97)	24.89^***^ (0.04)
*N*	5,808	5,808
Adjusted R^2^	0.08	0.09

2018 China Health and Retirement Longitudinal Study (CHARLS).

^+^*p* < 0.1, ^*^p < 0.05, ^**^p < 0.01, ^***^p < 0.001. Numbers in parentheses are standard errors.

We first conducted a series of analyzes to make sure that prerequisite requirements for applying the KHB method were met, i.e., the effects of independent variables on dependent variables, the effects of independent variables on mediating variables, and the effects of mediating variables on dependent variables were statistically significant. Since the three mediating variables were dichotomous, we conducted logistic regressions to determine if the two main independent variables had significant effects on the mediating variables. The results showed significantly positive effects of two main independent variables on all three mediating variables. Considering the dependent variables were all coded as continuous variables, we further performed OLS regression analyzes to investigate the effects of independent as well as mediating covariates on dependent variables. The results showed that the number of hours taking care of grandchildren had significantly positive effect on grandparents’ depressive level; and the impact of giving care to grandchildren on grandparents’ depression and life satisfaction also showed significantly positive effects (results were not shown here but are available upon request). Such findings suggested that the effects of independent variables on the respondent’s health outcomes were statistically significant. We then constructed regression models in Tables 3, 4 to test whether mediating effects existed when using grandchild caregiving and length of grandchild caregiving to predict grandparents’ health outcomes, respectively. If the regression coefficients in Model 1 changed in either values or significance levels when adding mediating variables in Model 2, then it indicated the mediating effects of internet use existed.

As Table 3 showed, when including mediating variables in Model 2, the regression coefficient for grandchild care predicting SRH reduced from 0.03 in Model 1 to 0.02 in Model 2, suggesting a significant effect of mediating variables when linking grandchild care to grandparents’ SRH. The regression coefficient for grandchild care predicting IADL changed from −0.42 in Model 1 to −0.32 in Model 2, which also suggested significant mediating effects of internet use variables. Similarly, the regression coefficient for grandchild care decreased from −0.20 in Model 1 to −0.18 in Model 2, evidencing significant mediating effects of internet use covariates. But the mediating effects were not shown when predicting life satisfaction. Table 4 showed that when including mediating variables to analyze grandparents’ health, the logistic regression coefficient for hours of giving grandchild care were 0.04 and 0.03 in Models 1 and 2, respectively. Such findings implied significant mediating effects. By performing the above analyzes, we identified the pathways that mediating variables showed significant effects. The next step of the study was to use the KHB method to detect total, direct and indirect effects to further explain what exact roles the internet use variables played in this study.

### 4.3. The mediating effects based on the KHB methods

Table 5 presented the results of running different mediation models for all respondents and for the four subgroups separately. The KHB method was used to decompose the total effect of internet use variables on grandparents’ health outcomes into indirect and (remaining) direct effects. Due to space constraint, regression coefficients were not presented for control variables. Also, only *significant* mediation effects were presented in Table 5. Since mediating effects were not found for the life satisfaction measure, models were only constructed for other three measures of health status. The Pseudo R-squared values were also presented in Table 5 to indicate goodness of fit of the models. As the table showed that when using grandchild caregiving as well as mediating variables to predict grandparental health, models predicting SRH had greater Pseudo R-squared values, indicating a better fit of the models when predicting SRH. The Pseudo R-squared value of 0.13 suggested that the regression model explains 13.0% variations of SRH. The Pseudo R-squared values for models predicting depression were the lowest, suggesting the model fit is not as good as predicting other health measures.

**Table 5:** Total, direct, and indirect effects of internet use on health outcomes, using the KHB method: Chinese grandparents aged 50–80.

Variables	Self-rated health			IADL			Depression		
	Total effect	Direct effect	Indirect effect	Total effect	Direct effect	Indirect effect	Total effect	Direct effect	Indirect effect
*All Respondents*									
X1: Cared for grandchild(ren)									
M1: Used internet to chat	–			−0.29^***^	−0.30^***^	0.01^*^	–		
M2: Used internet to watch news	–			–			−0.23^*^	−0.29^**^	0.06^***^
M3: Used internet to watch videos	0.07^*^	0.08*	−0.01^+^	–			–		
Pseudo R-squared	0.13			0.11			0.10		
X2: # of hours cared for grandchild(ren)/day									
M1:Used internet to watch news	–			–			0.05^***^	0.04^***^	0.01^*^
Pseudo R-squared	–			–			0.09		
*Subgroup1: (Urban Grandfathers)*									
X1: Cared for grandchild(ren)									
M1: Used internet to chat	–						–		
M2: Used internet to watch news	–			–			−0.18	−0.29	0.12^**^
M3: Used internet to watch videos				–			–		
Pseudo R-squared	–			–			0.08		
X2: # of hours cared for grandchild(ren)/day									
M1: Used internet to watch news	–			–			0.06^**^	0.06^**^	0.01^+^
Pseudo R-squared	–			–			0.09		
*Subgroup2 (Rural Grandfathers)*									
X1: Cared for grandchild(ren)									
M1: Used internet to chat	–			−0.23^**^	−0.22^*^	0.01^+^	–		
M2: Used internet to watch news	–			–			0.08	0.13	−0.04^*^
M3: Used internet to watch videos	0.04	0.05	−0.01^+^	–			–		
Pseudo R-squared	0.13			0.09			0.06		
X2: # of hours cared for grandchild(ren)/day									
Used internet to watch news	–			–			0.01	0.01	0.00
Pseudo R-squared	–			–			0.06		
*Subgroup3 (Urban Grandmothers)*									
X1: Cared for grandchild(ren)									
M1: Used internet to chat	–			−0.07	−0.10	0.03^*^	–		
M2: Used internet to watch news	–			–			0.45	0.31	0.14^**^
M3: Used internet to watch videos	0.02	−0.01	0.03^***^				–		
Pseudo R-squared	0.11			0.09			0.06		
X2: # of hours cared for grandchild(ren)/day									
M1: Used internet to watch news	–			–			0.08	0.07	0.01^+^
Pseudo R-squared	–			–			0.06		
*Subgroup4 (Rural Grandmothers)*									
X1: Cared for grandchild(ren)									
M1: Used internet to chat	–			−0.50^***^	−0.52^***^	0.02^*^	–		
M2: Used internet to watch news	–			–			−0.92^***^	−0.94^***^	0.02^***^
M3: Used internet to watch videos	0.07^*^	0.08^*^	−0.01	–			–		
Pseudo R-squared	0.10			0.12			0.06		
X2: # of hours cared for grandchild(ren)/day									
M1: Used internet to watch news	–			–			0.06^***^	0.05^***^	0.01^*^
Pseudo R-squared	–			–			0.05		

2018 China Health and Retirement Longitudinal Study (CHARLS).

^+^*p* < 0.1, ^*^p < 0.05, ^**^p < 0.01, ^***^p < 0.001. X refers to independent variable; M refers to mediating variable. Only significant mediating effects are presented in table.

The main findings drawn from Table 5 can be summarized as follows: When using grandparental caregiving variable along with mediating variables to predict SRH, the results showed that the total effect of caregiving experience on SRH was positive. This meant that giving care to grandchildren was beneficial to grandparents’ SRH. It increased the SRH score by 7.0% (e^(0.07)^-1 = 0.07) for the model that contained all respondents. By introducing the mediating variable, using internet to watch videos, the direct effect of the caregiving variable on SRH was 0.8, suggesting using internet to watch videos increased the positive effect of grandchild caregiving on health by 1.0% (e^(0.08–0.07)^-1 = 0.01). The mediating effect was found to be significant among rural grandfathers and rural grandmothers.

When using covariates to predict the IADL measure, having grandchild caregiving experience was found to be significantly negatively associated with grandparents’ IADL difficulty scores with the total effect being −0.29 in the model for all respondents. When including the mediating variable, using internet to chat, the direct effect was −0.30 and the indirect effect became 0.01. These values indicated that using internet to chat had a beneficial effect, which decreased the likelihood of grandparents having difficulties in instrumental activities of daily living by 1.0% (e^(−0.29 + 0.30)^–1 = 0.01) when using grandchild caregiving experience to predict it. Except for the subgroup of urban grandfathers, such a mediating effect was significant among all other three subgroups. The mediating effect of the internet usage variable was relatively stronger for urban grandmothers as compared to other subgroups.

**Figure 2:**
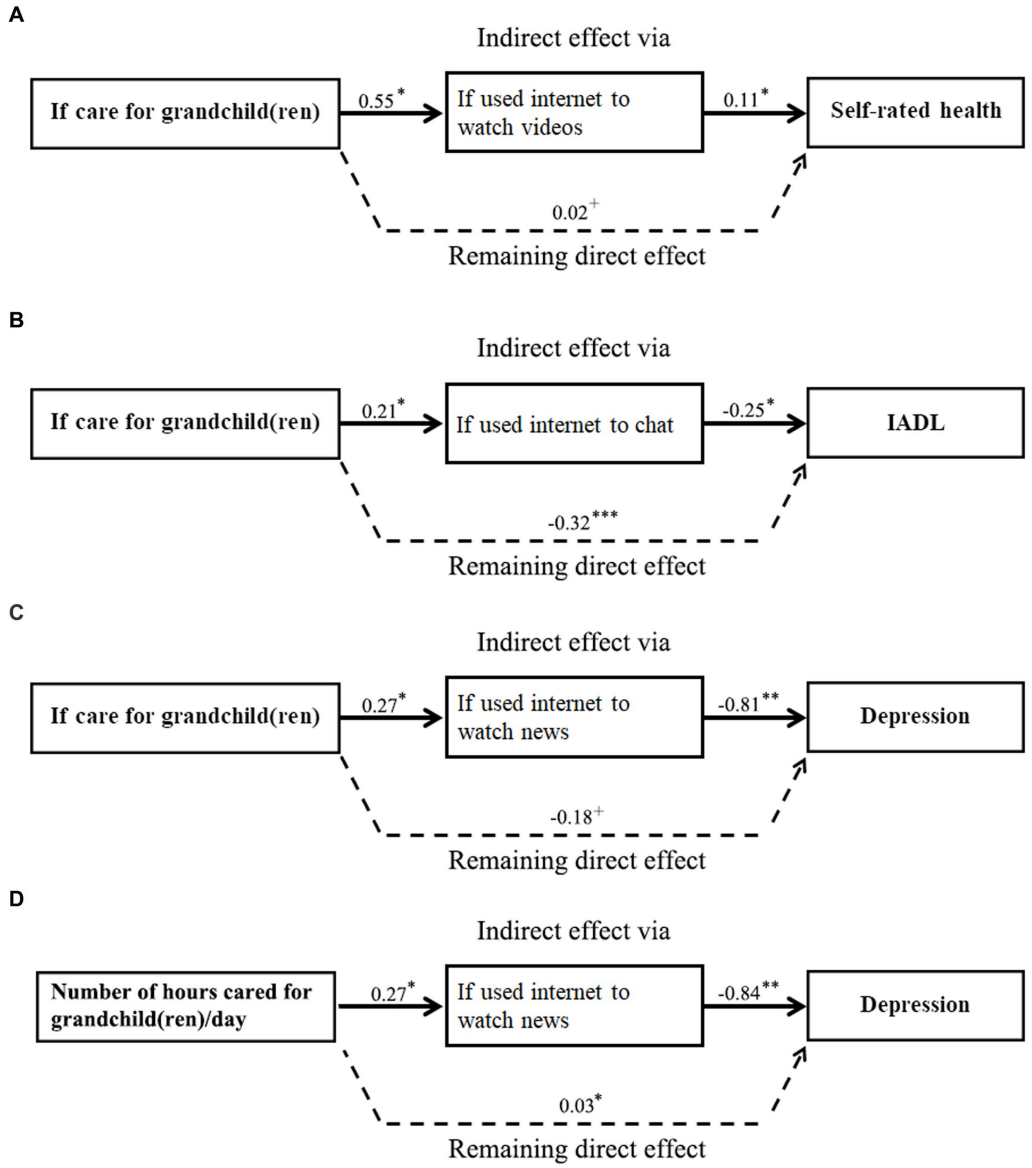
**(A–D)** Mediating effects indicating pathways through which grandparental childcare affects grandparents’ health. **(A)** Pathway through using internet to watch videos. **(B)** Pathway through using internet to chat. **(C)** Pathway through using internet to watch news. **(D)** Pathway through using internet to watch news.

Regarding depression, the results showed that using internet to watch news tended to be a pathway variable that mediated with both caregiving variables to impact grandparents’ depression level. For the model that studied all respondents, when the caregiving experience variable mediated with the watching news variable to predict grandparents’ depression, the total and direct effects were − 0.23 and − 0.29, respectively. The indirect effect of 0.06 implied that for those who cared for grandchild(ren), using internet to watch news further dropped grandparents’ depression level by 6.0% (e^(−0.23 + 0.29)^-1 = 0.06) when linked to grandparental caregiving. Similarly, when number of hours caring for grandchildren was used to predict grandparents’ depression, all three effects in the model were significant with indirect effect being 0.01. It suggested that longer hours of grandchild care increased grandparents’ depression level; internet usage offset the detrimental effect of long hours caregiving on the respondent’s mental health by 1.0%. The mediating effects were found in all four subgroups with the mediating effect being strongest among urban grandmothers. Figures 2A–D showed the pathways with significant mediating effects that were discussed above.

## 5. Discussion

Previous studies documented a strong relationship between grandparents caring for grandchildren and grandparental health (5, 11, 12, 26, 30). This research attempted to improve the existing literature by investigating the mediating effects of internet use in the linkage between grandparental child care and grandparents’ health status. The descriptive results showed that except for SRH, grandparents who had the experience of caring for at least one grandchild reported better mental health status, higher level of life satisfaction as well as lower IADL scores. Such findings were consistent with previous findings that offering care to grandchildren benefited grandparents’ health (30, 78).

Internet use was found to be a factor that mediated the relationship between caring for grandchildren and grandparents’ health. Specifically, watching videos promoted the beneficial effect of caregiving on grandparental health. Chatting through the internet decreased the negative effect of caregiving on IADL. Watching news through the internet was also found to decrease the negative consequence of caregiving on depression. In sum, internet use served as a mediator that promoted the positive effect of caregiving on grandparents’ health. We propose several rationales that may explain why internet use has played a mediating role in the link between intergenerational caregiving and grandparental health.

First, *social connectedness* grandparents gained through internet use offsets isolation that they may experience from childcare. As it was mentioned in earlier section of the paper, caregiving is time consuming, which prevents grandparents from social engagement and participating in leisure activities. As a result, heightened isolation and depression occur. Using the internet increases grandparents’ social communication and entertainment and offers new channels for older people to stay in contact or socialize with families and friends through messaging or chatgroups. It helps them to obtain new information, improve technical skills, and conduct commercial transactions. This rationale perhaps explains why watching news through the internet decreased the negative consequence of caregiving on depression found in this study. This is likely attributable to the fact that watching news makes grandparents feel less isolated from the society and more involved in social events. This rationale falls in line the *social causation theory* which emphasizes the importance of social networking and social engagement (41, 43).

Secondly, the internet can serve as a valuable source of support, which broadens grandparents’ social networks and helps older adults to fulfill their needs for companionship and overcome negative feelings and events including intergenerational conflicts (79). The support and sense of relief from online ties in turn benefit older adults’ health. Previous studies documented that the quality of grandparents’ relationships with their adult children and grandchildren are essential for their physical and mental health as well as subjective well-being (80). Evidence also indicated that older adults with weak intergenerational emotional cohesion are more likely to seek help outside the family, such as friendship and online ties (81). For the studied sample in this research, chatting through the internet may serve as an important way of social networking, which improved seniors’ functional independence dealing with daily activities and eased their frustration. This rationale is consistent with results presented in prior literature that social support mediated the influence of grandparental child care on grandparents’ health (55–57).

The third rationale relates to the *compensatory internet use theory* that values useful health-related information internet use brings to individuals (47, 48). Chinese grandparents tended to watch videos relating to longevity and health preservation. As researchers indicated, most internet users seek health related information from websites and discussion groups (82). Compared to other age groups, elders were especially interested in the internet for health related reasons (83). A study of Chinese sample showed that over 60.1% of the respondents claimed using internet to search health information (84). Therefore, practicing and internalizing healthy behaviors and lifestyles obtained from videos on the internet may explain the mediating effect of internet use.

The fourth rationale is drawn from the argument of *bonding social capital*. Bonding social capital refers to an individual’s close ties with families or friends. Internet has the advantages of convenience, connectivity, and ubiquity. Younger generation has become highly dependent on the Internet in daily lives, including online food ordering, shopping, banking, information seeking, leisure activities et al. (85). Being an internet user puts older adults in the same online contexts with their adult children. Internet use may help to resolve intergenerational conflicts between grandparents and their adult children by increasing the shared-identity bonds between two generations (86).

Internet use did not show a mediating effect on grandparents’ life satisfaction. Such results suggested that internet use may alleviate caregivers’ depression level and promote physical health, but it had limited influence on grandparents’ satisfaction toward life. This is probably attributable to the fact that when posting information on the website, people tended to upload photos, videos, stories that show positive sides of life. Receiving such information bring psychological loss and relative deprivation, which reduced people’s life satisfaction. This finding is consistent with *social comparison theory* (53, 54).

Besides investigating the mediating effects of internet use, another focus of the research was to explore the rural–urban and gender differences. Findings of the study painted a complex picture of the health implications. Overall, urban grandparents showed better physical and mental health than their rural counterparts with urban grandfathers reporting best health outcomes and rural grandmothers having worst health status. As to the gender differentials, grandfathers showed better health outcomes as compared to their female counterparts in both rural and urban settings. When the KHB method was applied to explore how caregiving and internet use shaped grandparental health, variations arose among subgroups as well. Clearly, watching videos through internet only showed a significant mediating effect on SRH among urban grandmothers and rural grandfathers. The mediating effect of using internet to chat on IADL was significant among all other three subgroups except for the urban grandfather group. Additionally, the mediating effect of using internet to watch news on depression was significant among all subgroups with the mediating effect being strongest among urban grandmother and grandfather subgroups. It is interesting to see that for some subgroups, the effects of caregiving and mediating variables were not consistent with the whole sample group. For example, watching videos reduced the positive effect of grandparenting on urban grandmothers’ SRH. Among rural grandfathers, grandparenting linked to a higher level of depression; watching news mediated with caregiving, which further increased caregivers’ depression level. For urban grandmothers, although watching news mediated with caregiving to reduce the depression level of caregivers, caregiving was found to be positively associated with grandmothers’ depression level. These findings implied that urban grandmothers somehow differed from other grandparents subgroups.

The gender differences shown in this study may be caused by gender differences in division of labor. Urban grandmothers provide more intensive care such as cooking, housekeeping, feeding, bathing, and dressing (11). Caregiving was therefore more demanding than rewarding for urban grandmothers. The gender differences may also be explained by the differentials between men and women in terms of sources and returns of social capital. Scholars documented that women are more likely to have a larger proportion of kin in their social networks, men tend to have more social capital from the workplace or formal organizations. Prior research found that informal socializing, such as socializing with family or friends, contributed more to female older adults’ life satisfaction and subjective wellbeing (87). Using the internet to maintain ties with families and friends helps older adults to foster the family cohesion, which enhances older Chinese women’s life satisfaction and health to a greater extent as compared to men (88). Previous studies also identified gender differences in internet use purposes and patterns. Females were found to use the internet more for communicative purposes, whereas males are more likely to use the internet for leisure activities (89). Female internet users are more likely to use the internet to connect with their adult children, especially for those left-behind grandmothers in rural areas who take care of grandchildren while their adult children migrated to cities for job opportunities. Thus, the parent–child contact through the internet is likely to be stronger among females than males and females benefit more from internet use than males.

## 6. Conclusion and implications

This study investigated the linkage between giving care to grandchildren and grandparents’ health in middle and later life, paying special attention to its mechanisms. Caregiving experience generally showed a positive effect on grandparents’ health. Such a finding is consistent with previous studies that giving care to grandchildren was beneficial to grandparents’ health (5, 8, 23). In addition to that linkage, the association was also found to be mediated by internet usage. All three internet use variables performed as mediators to either diminish the detrimental influence or increase the positive effect of caring giving on grandparental health. Thus far, previous analyzes have focused on documenting family cohesion, intergenerational relations, social networking, participating in social activities, and sleeping time as mediating factors which had either positive or negative indirect effects on grandparents’ health and level of life satisfaction (4, 5, 55–57). Hardly any studies have considered internet use as a mediator in the above linkage. Findings of this study shed light on the mediating effect of internet use in such an association, which enriched the existing literature and theories on mechanisms explaining grandparenting and grandparents’ health. The study also highlighted the urban–rural and gender differences, which reminded researchers to consider such differentials when studying grandparents’ health.

The study had limitations that need to be addressed in future research. One limitation was the relatively crude measures of caregiving. Detailed measures of intensive versus non-intensive care were not applied in this study. In addition, there could be recall bias in terms of grandparents’ self-reports of caregiving activities. Future research may rely on survey data that are collected by better survey instruments to measure caregiving. Another limitation related to the causality issue. Ideally, regressions should be conducted by analyzing the baseline and follow-up data of CHARLS. But the internet use variables were only available in the most recent wave, which prevented us from taking advantage of the longitudinal nature of the dataset to detect longitudinal changes in grandparenting and grandparental health. Thus, the current data has a limited capacity for adequately addressing issues of confounding or endogeneity in inferring causal relationships. Future research that covers a longer time span is warranted. Since the study mainly relied on studying the Chinese sample, findings may not be generalizable to other social contexts. Readers should interpret the results with caution.

Despite these limitations, this study had important theoretical contributions as well as practical implications. Theoretically, the study contributed to the literature on mechanisms of how caring for grandchildren links to grandparental health in a non-Western context. Previous studies have rarely paid attention to internet use as a pathway explaining grandparental health changes due to intergenerational caregiving. Results drawn from this research indicated that internet can serve as a mediator that improves grandparents’ health and reduces their depressive symptoms. Thus, findings of this study enriched existing theories on grandparenting and grandparents’ health. The study also has important practical implications. Findings of this research suggested that internet use may act as potential empowerment for older adults to maintain the connection with the outside world, which plays a critical role of benefiting older adults’ health and subjective well-being. When smartphones has become a predominant information source and an important communication channel for people, the digital divide is apparent in China, especially gray digital divide. It refers to the gaps in the prevalence, accessibility, literacy, and consequences of using digital devices between the younger and older generations (90). The rate of internet use among Chinese older adults is still low. By the end of 2018, people aged 60 and above accounted for 17.8% of the total population. Internet users aged 60 and above only accounted for only 5.2% of the total population in 2018 and the rate increased to 6.7% in 2020. The percentage of older adults using internet in rural areas is even lower (88). Accordingly, when policy makers and health professionals encourage grandparents accessing to the internet to improve their health status, they should focus on encouraging rural grandparents to access internet by using smartphones. The massive rural to urban migration has caused physical separation between older people and their adult children in rural China. A large amount of rural grandparents stay in skipped-generation households taking care of their grandchildren. They experience more difficulties in caregiving and more disadvantages in internet use due to limited accessibility and literacy to modern technology. Hence, it is urgent to increase internet accessibility among older adults, especially those in rural areas. They should be helped to learn how to use modern technology. This study also offered empirical evidence showing the gender differences between female and male grandparents in health status as well as internet use. In sum, the rural–urban and gender differentials call for more research to shed light on the benefits of internet use among subgroups of grandparents. Future policies also need to be tailored based on the heterogeneous features of Chinese grandparents’ subpopulations.

## Data availability statement

Publicly available datasets were analyzed in this study. This data can be found at: https://charls.pku.edu.cn/.

## Ethics statement

All participants signed an informed consent form and agreed to participate in the survey.

## Author contributions

LiZ and JW were major contributors. LiZ designed the research, conducted literature review, analyzed data and wrote the original draft. JW conducted literature review, formal analysis and revised the manuscript. RG and LaZ analyzed and interpreted the data. All authors read and approved the final manuscript.

## Funding

This research is supported by the Fundamental Research Funds for Central Universities (Grant Nos. 19ZFY84001 and 20ZFG84001). It is also funded by National Social Science Foundation of China (Grant No. 21BRK043) and the Distinguished Qian Duansheng Scholar Funds at China University of Political Science and Law. The funders had no role in the design of the study, in collection, analysis and interpretation of data, and in writing the manuscript.

## Conflict of interest

The authors declare that the research was conducted in the absence of any commercial or financial relationships that could be construed as a potential conflict of interest.

## Publisher’s note

All claims expressed in this article are solely those of the authors and do not necessarily represent those of their affiliated organizations, or those of the publisher, the editors and the reviewers. Any product that may be evaluated in this article, or claim that may be made by its manufacturer, is not guaranteed or endorsed by the publisher.

## References

[1] Burnette D Sun J Sun F. A comparative review of grandparent Care of Children in the U.S. and China. Ageing Int. (2013) 38:43–57. doi: 10.1007/s12126-012-9174-z, PMID: 36360724

[2] Chen F Short SE Entwisle B. The impact of grandparental proximity on maternal childcare in China. Popul Res Policy Rev. (2000) 19:571–90. doi: 10.1023/A:1010618302144

[3] Short SE Zhai F Xu S Yang M. China's one-child policy and the Care of Children: an analysis of qualitative and quantitative data. Soc Forces. (2001) 79:913–43. doi: 10.1353/sof.2001.0025, PMID: 29980230

[4] Shen LL Zhang Z. The Grandparenting and mental health of middle aged and elderly people: the mediating effect of family cohesion. Stud Psychol Behav. (In Chinese). (2020) 18:234–40. Available at: https://kns.cnki.net/kcms/detail/detail.aspx?FileName=CLXW202002013&DbName=CJFQ2020

[5] He QH Tan YF Peng ZC. How does grandchild care affect the health of grandparents?-new evidence from CHARLS. Population Develop. (In Chinese). (2021) 27:52–64. Available at: https://kns.cnki.net/kcms/detail/detail.aspx?FileName=SCRK202102005&DbName=CJFQ2021

[6] Lou V. Life satisfaction of Chinese grandmothers: the impact of Grandparenting role changes. J Ethn Cult Divers Soc Work. (2011) 20:185–202. doi: 10.1080/15313204.2011.594992

[7] Cong Z Silverstein M. Intergenerational time-for-money exchanges in rural China: does reciprocity reduce depressive symptoms of older grandparents? Res Hum Dev. (2008) 5:6–25. doi: 10.1080/15427600701853749

[8] Chen F Liu G Mair CA. Intergenerational ties in context: grandparents caring for grandchildren in China. Soc Forces. (2011) 90:571–94. doi: 10.1093/sf/sor012, PMID: 22544978 PMC3337769

[9] Xie X Xia Y. Grandparenting in Chinese immigrant families. Marriage Fam Rev. (2011) 47:383–96. doi: 10.1080/01494929.2011.594218, PMID: 30256661

[10] Xu H Liu J Zhang Z Li L. Sandwiched grandparents and biological health risks in China. J Health Soc Behav. (2022) 63:410–27. doi: 10.1177/00221465211069895 35012397

[11] Xu H. Physical and mental health of Chinese grandparents caring for grandchildren and great-grandparents. Soc Sci Med. (2019) 229:106–16. doi: 10.1016/j.socscimed.2018.05.047, PMID: 29866373 PMC6261790

[12] Di Gessa G Glaser K Tinker A. The impact of caring for grandchildren on the health of grandparents in Europe: a lifecourse approach. Soc Sci Med. (2016) 152:166–75. doi: 10.1016/j.socscimed.2016.01.041, PMID: 26854626

[13] Blustein J Chan S Guanais FC. Elevated depressive symptoms among caregiving grandparents. Health Serv Res. (2004) 39:1671–90. doi: 10.1111/j.1475-6773.2004.00312.x, PMID: 15533181 PMC1361092

[14] Szinovacz ME DeViney S Atkinson MP. Effects of surrogate parenting on Grandparents' well-being. J Gerontol B Psychol Series B. (1999) 54B:S376–88. doi: 10.1093/geronb/54B.6.S376, PMID: 10625973

[15] Yang H Zhang S Zhang SQ Xie L Wu Y Yao Y. Internet use and depressive symptoms among older adults in China. Front Public Health. (2021) 12:739085. doi: 10.3389/fpsyt.2021.739085 PMC8688754 34950065

[16] Li LW Liu J Xu H Zhang Z. Understanding rural-urban differences in depressive symptoms among older adults in China. J Aging Health. (2016) 28:341–62. doi: 10.1177/0898264315591003, PMID: 26100620 PMC4893784

[17] Meisner M. Mao's China and After: A History of the People's Republic. 3rd ed. New York: Free Press (1999).

[18] Wu X Treiman DJ. The household registration system and social stratification in China: 1955-1996. Demography. (2004) 41:363–84. doi: 10.1353/dem.2004.0010, PMID: 15209045

[19] Yang S. Rural and Urban Daily Life: A Sociological Analysis. Beijing: China Social Sciences Press (2008).

[20] Zimmer Z Kwong J. Socioeconomic status and health among older adults in rural and urban China. J Aging Health. (2004) 16:44–70. doi: 10.1177/0898264303260440, PMID: 14979310

[21] Giles J Wang D Zhao C. Can China’s rural elderly count on support from adult children? Implications of rural-to-urban migration. J Population Ageing. (2011) 3:183–204. doi: 10.1007/s12062-011-9036-6

[22] Yu J Xie Y. Cohabitation in China: trends and determinants. Popul Dev Rev. (2015) 41:607–28. doi: 10.1111/j.1728-4457.2015.00087.x, PMID: 30416225 PMC6226097

[23] Chen F Liu G. The health implications of grandparents caring for grandchildren in China. J Gerontol. (2012) 67:99–112. doi: 10.1093/geronb/gbr132, PMID: 22156630 PMC3267025

[24] Jang H Tang FY. Effects of social support and volunteering on depression among grandparents raising grandchildren. Int J Aging Hum Dev. (2016) 83:491–507. doi: 10.1177/0091415016657561 27418590

[25] Baker LA Merril S. Preventive health behaviors among grandmothers raising grandchildren. J Gerontol B Psychol Sci Soc Sci. (2008) 63:S304–11. doi: 10.1093/geronb/63.5.S304 18818451 PMC2633920

[26] Ates M. Does grandchild care influence grandparents' self-rated health? Evidence from a fixed effects approach. Soc Sci Med. (2017) 190:67–74. doi: 10.1016/j.socscimed.2017.08.021, PMID: 28843872

[27] Waldrop DP Weber JA. From grandparent to caregiver: the stress and satisfaction of raising grandchildren. Fam Soc. (2001) 82:461–72. doi: 10.1606/1044-3894.177, PMID: 35860992

[28] Kelley SJ Whitley DM Campos PE. Psychological distress in African American grandmothers raising grandchildren: the contribution of child behavior problems, physical health, and family resources. Res Nurs Health. (2013) 36:373–85. doi: 10.1002/nur.21542, PMID: 23606233

[29] Peng Y Chan Y. Do internet users Lead a healthier lifestyle? J Appl Gerontol. (2020) 39:277–84. doi: 10.1177/0733464818785797, PMID: 29957087

[30] Arpino B Bordone V. Does grandparenting pay off? The effect of child care on grandparents’ cognitive functioning. J Marriage Fam. (2014) 76:337–51. doi: 10.1111/jomf.12096

[31] Engelhardt H Buber I Skirbekk V Prskawetz A. Social involvement, behavioural risks and cognitive functioning among older people. Ageing Soc. (2010) 30:779–809. doi: 10.1017/S0144686X09990626

[32] Hilbrand S Coall DA Meyer AH Gerstorf D Hertwig RA. Prospective study of associations among helping, health, and longevity. Soc Sci Med. (2017) 187:109–17. doi: 10.1016/j.socscimed.2017.06.035, PMID: 28683378

[33] Tsai F-J Motamed S Rougemont A. The protective effect of taking care of grandchildren on elders' mental health? Associations between changing patterns of intergenerational exchanges and the reduction of elders' loneliness and depression between 1993 and 2007 in Taiwan. BMC Public Health. (2013) 13:1043969. doi: 10.1186/1471-2458-13-567, PMID: 23758624 PMC3689038

[34] Ku L-J Stearns S Van Houtven C Lee S-Y Dilworth-Anderson P Konrad T. Impact of caring for grandchildren on the health of grandparents in Taiwan. J Gerontol B. (2013) 68:1009–21. doi: 10.1093/geronb/gbt090, PMID: 24056691

[35] Xu L Wu B Chi I Hsiao H-Y. Intensity of grandparent caregiving and life satisfaction among rural Chinese older adults: a longitudinal study using latent difference score analysis. Fam Community Health. (2012) 35:287–99. doi: 10.1097/FCH.0b013e31826665d0, PMID: 22929375

[36] Hilbrand S Coall AD Gerstorf D Hertwig R. Caregiving within and beyond the family is associated with lower mortality for the caregiver. Evol Hum Behav. (2017) 38:397–403. doi: 10.1016/j.evolhumbehav.2016.11.010, PMID: 30212122

[37] Cong Z Silverstein M. Caring for grandchildren and intergenerational support in rural China: a gendered extended family perspective. Ageing Soc. (2012) 32:425–50. doi: 10.1017/S0144686X11000420

[38] Hughes ME Waite LJ LaPierre TA Luo Y. All in the family: the impact of caring for grandchildren on grandparents’ health. J Gerontol B Psychol Sci Soc Sci. (2007) 62:S108–19. doi: 10.1093/geronb/62.2.S108, PMID: 17379680 PMC2562755

[39] Oshio T. Is caring for grandchildren good for grandparents’ health? Evidence from a fourteen-wave Nationwide survey in Japan. J Epidemiol. (2022) 32:363–9. doi: 10.2188/jea.JE20200529, PMID: 33518593 PMC9263614

[40] Glaser K di Gessa G Tinker A. Grandparenting in Europe. The Health and Wellbeing of Grandparents Caring for Grandchildren: The Role of Cumulative Advantage/Disadvantage. London: Grandparents Plus (2014).

[41] Hampton KN. Social media and change in psychological distress over time: the role of social causation. J Comput-Mediat Commun. (2019) 24:205–22. doi: 10.1093/jcmc/zmz010

[42] Heo J Chun S Lee S Lee KH Kim J. Internet use and well-being in older adults. Cyberpsychology Behav Soc Netw. (2015) 18:268–72. doi: 10.1089/cyber.2014.0549, PMID: 25919967

[43] Chen YRR Schulz PJ. The effect of information communication technology interventions on reducing social isolation in the elderly: a systematic review. J Med Internet Res. (2016) 18:1–11. doi: 10.2196/jmir.4596 PMC4751336 26822073

[44] Yu RP Mccammon RJ Ellison NB Langa KM. The relationships that matter: social network site use and social wellbeing among older adults in the United States of America. Ageing Soc. (2016) 36:1826–52. doi: 10.1017/S0144686X15000677, PMID: 31356148

[45] Plaza I Martín L Martin S Medrano C. Mobile applications in an aging society: status and trends. J Syst Softw. (2011) 84:1977–88. doi: 10.1016/j.jss.2011.05.035

[46] Veena C Kwon W Juan G. Internet use and perceived impact on quality of life among older adults: a phenomenological investigation. Int J Health. (2012) 2:1–13. doi: 10.18848/2156-8960/CGP/v02i03/41021

[47] Tsai HYS Shillair R Cotten S Winstead V Yost E. Getting grandma online: are tablets the answer for increasing digital inclusion for older adults in the U.S.? Educ Gerontol. (2015) 41:695–709. doi: 10.1080/03601277.2015.1048165, PMID: 26877583 PMC4748942

[48] Joe J Demiris G. Older adults and mobile phones for health: a review. J Biomed Inform. (2013) 46:947–54. doi: 10.1016/j.jbi.2013.06.008, PMID: 23810858 PMC3836587

[49] Lee RYW Carlisle AJ. Detection of falls using accelerometers and mobile phone technology. Age Ageing. (2011) 40:690–6. doi: 10.1093/ageing/afr050, PMID: 21596711

[50] Muusses LD Finkenauer C Kerkhof P Billedo CJ. A longitudinal study of the association between compulsive internet use and wellbeing. Comput Hum Behav. (2014) 36:21–8. doi: 10.1016/j.chb.2014.03.035, PMID: 36334225

[51] Marttila E Koivula A Rasanen P. Does excessive social media use decrease subjective well-being? A longitudinal analysis of the relationship between problematic use, loneliness and life satisfaction. Telematics Inform. (2021) 59:101556. doi: 10.1016/j.tele.2020.101556

[52] Chou HTG Edge N. “They are happier and having better lives than I am”: the impact of using Facebook on perceptions of others' lives. Cyberpsychol Behav Soc Netw. (2012) 15:117–21. doi: 10.1089/cyber.2011.0324, PMID: 22165917

[53] Verduyn P Lee DS Park J. Passive Facebook usage undermines affective well- being: experimental and longitudinal evidence. J Exp Psychol. (2015) 144:480–8. doi: 10.1037/xge0000057, PMID: 25706656

[54] Frison E Eggermont S. Exploring the relationships between different types of Facebook use, perceived online social support, and adolescents depressed mood. Soc Sci Comput Rev. (2015) 34:153–71. doi: 10.1177/0894439314567449

[55] Tang D Sun H Xu Y. The impacts of grandchild care on mental health among Chinese older adults: the mediating effects of social networks. Population Res (in Chinese). (2020) 44:33–45. Available at: https://kns.cnki.net/kcms/detail/detail.aspx?FileName=RKYZ202004003&DbName=CJFQ2020

[56] Zhou J Mao W Lee Y Chi I. The impact of caring for grandchildren on grandparents’ physical health outcomes: the role of intergenerational support. Res Aging. (2017) 39:612–34. doi: 10.1177/0164027515623332, PMID: 26733495

[57] Noriega C Velasco C Pérez-Rojo G López J. Character strengths and social support as protective factors between Grandparents' caregiving and health-related quality of life. J Child Fam Stud. (2022) 31:2505–17. doi: 10.1007/s10826-021-02187-9

[58] Jolie C Lam B. Digital inclusiveness--longitudinal study of internet adoption by older adults. J Manag Inf Syst. (2006) 22:177–206. doi: 10.2753/MIS0742-1222220407

[59] Swickert R Hittner J. Relationship among internet use, personality, and social support of the elderly. Comput Hum Behav. (2002) 18:437–51. doi: 10.1016/S0747-5632(01)00054-1, PMID: 37444058

[60] Zhang Q Li Z. The impact of internet use on the social networks of the elderly in China-the mediating effect of social participation. Int J Environ Res Public Health. (2022) 19:9576. doi: 10.3390/ijerph19159576, PMID: 35954933 PMC9367896

[61] Li J. Research on Culture of Contemporary Chinese Urban Elders. Shanghai: Shanghai People's Press (2012).

[62] Lv M Zhang Y. Does internet use help elderly people get intergenerational support?-heterogeneity research based on urban and rural dimensions. World Survey Res (In Chinese). (2021) 7:35–45. doi: 10.13778/j.cnki.11-3705/c.2022.07.004

[63] Martinez-Gomez D Guallar-Castillon P Garcia-Esquinas E Bandinelli S Rodriguez-Artalejo F. Physical activity and the effect of multimorbidity on all-cause mortality in older adults. Mayo Clin Proc. (2017) 92:376–82. doi: 10.1016/j.mayocp.2016.12.004, PMID: 28160946

[64] Smith K Victor C. Typologies of loneliness, living alone and social isolation, and their associations with physical and mental health. Ageing Soc. (2019) 39:1709–30. doi: 10.1017/S0144686X18000132

[65] Ferraro K Schafer M Wilkinson L. Childhood disadvantage and health problems in middle and later life: early imprints on physical health? Am Sociol Rev. (2016) 81:107–33. doi: 10.1177/0003122415619617, PMID: 27445413 PMC4950981

[66] Meyer OL Castro-Schilo L Aguilar-Gaxiola S. Determinants of mental health and self-rated health: a model of socioeconomic status, neighborhood safety, and physical activity. Am J Public Health. (2014) 104:1734–41. doi: 10.2105/AJPH.2014.302003, PMID: 25033151 PMC4151927

[67] Li Y Xu L Chi I Guo P. Participation in productive activities and health outcomes among older adults in urban China. The Gerontologist. (2013) 54:784–96. doi: 10.1093/geront/gnt106, PMID: 24030035

[68] Peeters GM Verweij LM van Schoor NM Pijnappels M Pluijm SM Visser M. Which types of activities are associated with risk of recurrent falling in older persons? J Gerontol Ser A Biol Med Sci. (2010) 65:743–50. doi: 10.1093/gerona/glq013, PMID: 20159779

[69] Wang SQ Ying J Zhang ML Shi Y Li Y Xing ZJ. Health-related life satisfaction and its influencing factors: a cross-sectional study in China. Jpn J Nurs Sci. (2018) 15:285–97. doi: 10.1111/jjns.12201 29363255

[70] Yang H Wu Y Lin X Xie L Zhang S Zhang S. Internet use, life satisfaction, and subjective well-being among the elderly: evidence from 2017 China general social survey. Front Public Health. (2021) 9:677643. doi: 10.3389/fpubh.2021.677643, PMID: 34268289 PMC8275954

[71] Zhu Y Guo X Zhang X Shi X Yang Y Zhang Q. Sex differences in the relationship of serum creatinine to cystatin C ratio and depressive symptoms among middle-aged and older adults in China. J Affect Disord. (2022) 319:57–61. doi: 10.1016/j.jad.2022.09.030, PMID: 36116601

[72] Li LW Liu J Zhang Z Xu H. Late-life depression in rural China: do village infrastructure and availability of community resources matter? Int J Geriatr Psychiatry. (2015) 30:729–36. doi: 10.1002/gps.4217, PMID: 25333218 PMC4710465

[73] Glorioso V Pisati M. Socioeconomic inequality in health-related behaviors: a lifestyle approach. Qual Quant. (2014) 48:2859–79. doi: 10.1007/s11135-013-9929-y, PMID: 36199109

[74] Xu H Xie Y. Socioeconomic inequalities in health in China: a reassessment with data from the 2010-2012 China family panel studies. Soc Indic Res. (2017) 132:219–39. doi: 10.1007/s11205-016-1244-2, PMID: 28694561 PMC5501396

[75] Breen R Karlson KB Holm A. A note on a reformulation of the KHB method. Sociol Methods Res. (2018) 50:901–12. doi: 10.1177/0049124118789717

[76] Kohler U. Comparing coefficients of nested nonlinear probability models using khb. Stata J. (2011) 11:420–38. doi: 10.1177/1536867X1101100306

[77] van den Berg L Kalmijn M Leopold T. Family structure and early home leaving: a mediation analysis. European J Population. (2018) 34:873–900. doi: 10.1007/s10680-017-9461-1, PMID: 30976265 PMC6261855

[78] Hilbrand S Coall DA Meyer AH Gerstorf D Hertwig R. A prospective study of associations among helping, health, and longevity. Soc Sci Med. (2017) 187:109–17. doi: 10.1016/j.socscimed.2017.06.035, PMID: 28683378

[79] Morahan-Martin J Schumacher P. Loneliness and social uses of the internet. Comput Hum Behav. (2003) 19:659–71. doi: 10.1016/S0747-5632(03)00040-2, PMID: 37260952

[80] Coimbra S Mendonca MG. Intergenerational solidarity and satisfaction with life: mediation effects with emerging adults. Paidéia. (2013) 23:161–9. doi: 10.1590/1982-43272355201303

[81] Zhang X Silverstein M. Intergenerational emotional cohesion and psychological well-being of older adults in rural China: a moderated mediation model of loneliness and friendship ties. J Gerontol B Psychol Sci Soc Sci. (2022) 77:525–35. doi: 10.1093/geronb/gbab122, PMID: 34214164 PMC8893140

[82] Wang T Zhou X Ni Y Pan Z. Health information needs regarding diabetes mellitus in China: an internet-based analysis. BMC Public Health. (2020) 20:990. doi: 10.1186/s12889-020-09132-3, PMID: 32576159 PMC7310401

[83] Jia W. Diabetes: a challenge for China in the 21st century. Lancet Diabetes Endocrino. (2014) 2:e6–7. doi: 10.1016/S2213-8587(14)70027-0, PMID: 24703055

[84] Liang X Xiong F Xie F. The effect of smartphones on the self-rated health levels of the elderly. BMC Public Health. (2022) 22:508. doi: 10.1186/s12889-022-12952-0, PMID: 35292010 PMC8925210

[85] Huang H Zhang X. The adoption and use of WeChat among middle-aged residents in urban China. Chinese J Commun. (2017) 10:134–56. doi: 10.1080/17544750.2016.1211545

[86] Nyqvist F Forsman AK Giuntoli G Cattan M. Social capital as a resource for mental well-being in older people: a systematic review. Aging Ment Health. (2013) 17:394–410. doi: 10.1080/13607863.2012.742490, PMID: 23186534

[87] Kroll C. Different things make different people happy: examining social capital and subjective well-being by gender and parental status. Soc Indic Res. (2011) 104:157–77. doi: 10.1007/s11205-010-9733-1

[88] Li J Zhou X. Internet use and Chinese older adults’ subjective well-being (SWB): the role of parent-child contact and relationship. Comput Hum Behav. (2021) 119:106725. doi: 10.1016/j.chb.2021.106725

[89] Weiser EB. Gender differences in internet use patterns and internet application preferences: a two-sample comparison. CyberPsychol Behav. (2000) 3:167–78. doi: 10.1089/109493100316012

[90] Huxhold O Hees E Webster NJ. Towards bridging the grey digital divide: changes in internet access and its predictors from 2002 to 2014 in Germany. Eur J Ageing. (2020) 17:271–80. doi: 10.1007/s10433-020-00552-z, PMID: 32904732 PMC7459052

